# Depression and anxiety mediate the relationship between the retrospectively measured symptoms of premenstrual disorders and negative but not positive psychotic-like experiences

**DOI:** 10.1038/s41598-023-44573-x

**Published:** 2023-10-18

**Authors:** Rachela Antosz-Rekucka, Katarzyna Prochwicz

**Affiliations:** 1 Doctoral School in the Social Sciences, Jagiellonian University, Kraków, Poland; 2 Institute of Psychology, Jagiellonian University, Kraków, Poland

**Keywords:** Psychology, Human behaviour

## Abstract

The aim of this study was to examine the relationship between retrospectively measured premenstrual symptoms and subclinical forms of positive and negative psychotic symptoms (psychotic-like experiences; PLEs). It was hypothesised that subjective intensity of the symptoms of premenstrual disorders predicts PLEs frequency and that this relationship is mediated by anxiety and depression. The study sample comprised 108 non-clinical subjects. Study variables were assessed with self-report questionnaires: the Premenstrual Symptoms Screening Tool (PSST); the Beck Depression Inventory Second Edition (BDI-II); the State-Trait Anxiety Inventory (STAI; trait sub-scale); the Community Assessment of Psychic Experiences (CAPE). Regression and mediation analyses were performed. The PSST scores were significantly and positively associated with psychotic-like experiences frequency. The relation was stronger for positive PLEs. Anxiety and depression partially mediated the relationship between premenstrual symptoms and negative PLEs, but not between premenstrual symptoms and positive PLEs. Although the design of the study does not allow to infer causality, it demonstrates strong, positive relationship between the symptoms of premenstrual disorders and psychotic-like experiences. The relationship between premenstrual disorders and negative PLEs seems to be partially based on a general psychopathological factor. Further longitudinal studies are needed to test whether premenstrual disturbances increase risk of psychotic symptoms.

## Introduction

Premenstrual symptomatology can be seen as a continuum, ranging from none or mild symptoms, through premenstrual syndrome (PMS), to the severe manifestation of premenstrual disturbances: premenstrual dysphoric disorder (PMDD)^1,2^. Clinical PMS differs from ailments occurring in healthy menstruating people in terms of quantity (a set of symptoms), intensity (at least moderate) and impact on life (at least moderate functional impairment). Premenstrual dysphoric disorder is a severe form of PMS, characterised by high intensity of symptoms and significant functional impairment. Moreover, for PMDD to be diagnosed, the patient must present at least five out of 11 symptoms, one of which must be either depressed mood, increased irritability or anger, mood lability, or anxiety—the core emotional symptoms of PMDD—in a prospective measure of two consecutive menstrual cycles^3^. Other, so-called secondary symptoms include feelings of losing control, decreased interest in everyday activities, subjective problems with concentration, lethargy or lack of energy, overeating or food cravings, hyper- or insomnia, and somatic ailments such as breast tenderness or joint and muscle pain^3^. Symptoms must occur only in the late luteal phase of the cycle (between ovulation and menstruation, last week) and withdraw within a few days after menses.

PMDD happens in 3–8% of naturally-cycling individuals^4^ and clinical PMS is observed in about 20–30% of them^5^. Premenstrual disorders are related to decreased life quality, interpersonal conflicts, relational problems, lower education achievements and work productivity, lower sexual satisfaction, decreased engagement in social activities and hobbies^6–8^.

The psychosis continuum hypothesis assumes that states resembling psychotic symptoms occur among the healthy population^9^ and clinical cases constitute only a minor part of the psychosis continuum^10^. This approach challenges strong distinction between health and psychopathology. So-called psychotic-like experiences (PLEs) are subclinical symptoms of psychosis which are characterized by lower intensity and frequency, lower negative impact on life, lower intrusiveness and rigidity than full-blown psychosis^9^. They are often conceptualized as psychotic symptoms in the absence of illness^10^. In other words, PLEs differs from the full-blown psychosis in terms of quantity, not quality. Psychotic-like experiences can be divided into two subtypes, corresponding to the types of psychotic symptoms: positive (hallucination-like experiences and delusion-like beliefs) and negative (symptoms such as apathy, anhedonia, emotional blunting, social withdrawal, etc.). A meta-analysis by van Os et al.^9^ showed the prevalence of psychotic-like experiences at the level of 8% in the general population.

PLEs share many similarities with psychotic disorders, including risk factors (e.g., trauma, lower socioeconomic status, minority status, being single or divorced, substance abuse, living in a large city)^10,11^, gender differences (more positive and affective symptoms in women, more negative and disorganization symptoms in men)^12,13^, cognitive dysfunctions (e.g. cognitive biases)^14^ and emotional disturbances, depressive mood and anxiety in particular^15–17^.

Most PLEs are transient, although intensive, frequent, and persistent psychotic-like experiences constitute a risk factor for psychotic disorders^10,18^ as well as other psychiatric conditions, including mood and anxiety disorders^15,19^. Not only are PLEs correlated with distress and depressive symptoms^20^, but the diagnosis of depression or anxiety disorder was shown to be related with higher probability of experiencing PLEs^21^ and PLEs can predict depression and anxiety levels in three years^15^. Depression and anxiety were also shown to mediate the relationship between personality risk factors and PLEs^22^.

Although, to our knowledge, the relationship between premenstrual disorders and PLEs was not examined, there are some premises to predict that premenstrual disorders and psychotic symptoms may be related. Ullah et al.^23^ suggested that schizophrenia and PMDD may share some genetic basis and biomarkers. In German and Chinese cohorts both PMDD and schizophrenia were associated with copy number variations in a gene encoding GABAergic receptors (GABRB2)^23^. Moreover, the case study of Hsiao and Liu^24^ showed that delusions and auditory hallucinations may be unusual manifestations of PMS. The patients examined presented patterns of positive psychotic symptoms (including bewildering auditory hallucinations and reference delusions) occurring only in the last week of the luteal phase and resolving within a few days after menses^24^. It is hypothesised that reproductive hormones, especially estradiol, may affect a course of psychotic disorders^25,26^ and a significant increase in admission to the psychiatric hospital due to psychosis during the perimenstrual phase was observed in some studies^25,26^ and confirmed in a meta-analysis^27^. The hypothesised mechanism of that phenomenon is estradiol withdrawal in the perimenstrual phase, as estrogens seem to have a protective role against psychosis (but other reproductive hormones may also play a role)^26^.

It is also important to note that while a few psychotic and DSM-5 symptoms of PMDD may overlap (social withdrawal, lack of energy or motivation to engage in activities), traditionally psychotic or psychotic-like experiences are not included among premenstrual symptoms.

## Objectives

The current study was based on retrospective data. While this is not considered a fully reliable diagnosis of premenstrual disorders, the results of a retrospective measure can be treated as the indication of the presence of some premenstrual symptoms and a person’s beliefs or memories about own premenstrual ailments. We decided to call this phenomenon “the subjective intensity of symptoms”.

Based on the preliminarily findings of Hsiao and Liu^24^ and in accordance with the studies highlighting the association between menstruation cycle and the course of psychosis, we hypothesized that frequency of psychotic-like experiences is predicted by subjective intensity of premenstrual symptoms (that is retrospectively measured symptoms). Since core emotional symptoms of premenstrual disorders^3^, especially depressive mood and anxiety, are occurring commonly in PLEs^28^ and play an important role in delusions formation^29^ we assumed that these symptoms are crucial for the relationship between PMDD and PLEs. It is plausible that the increase of anxiety and depression before and during menstruation^30^ may contribute to escalation of psychotic symptoms^31,32^. Thus, in the current study depressive symptoms and trait anxiety were tested as potential mediators linking premenstrual disorders and PLEs. In summary, we hypothesise that the subjective intensity of premenstrual symptoms (expressed by the scores on a screening questionnaire) predicts the frequency of psychotic-like experiences and that depression and the said relationship is mediated by depression and trait anxiety.

## Methods

### Participants

The initial study sample consisted of 178 people (all white; 167 female, 9 non-binary, one transgender man, one did not specify their gender) recruited from university sample, from various faculties. Their age ranged from 18 to 25 (*M* = 20.73; *SD* = 1.54). All participants were volunteers and did not receive any compensation.

However, for higher relatability of results, some participants were excluded from analysis. The exclusion criteria were: (1) lack of regular menstruation cycles; (2) oral contraceptives usage, pregnancy, or serious medical condition; (3) usage of psychoactive substances (other than alcohol and nicotine) less than three months before the study (effects of the substance might be mistaken as PLEs); (4) declaration ongoing mental disorder diagnosis (some psychopathological symptoms might be mistaken as PLEs and premenstrual exacerbation of preexisting mental disorder could be mistaken with PMS or PMDD); (5) more than 5% incomplete answers.

The final sample consisted of 108 people (102 females, 6 non-binary people; aged 18–25, *M* = 20.91; *SD* = 1.65). The calculation of the sample size large enough to detect the mediated effect was based on Fritz and MacKinnon^33^ recommendations and as well as the G*Power tool^34^ and that sample size was established to be between 70 and 80 people at least. Thus, the final sample was sufficient to perform the analysis.

### Materials and procedure

Premenstrual symptoms were measured with the Polish version of the Premenstrual Symptoms Screening Tool (PSST)^35^. PSST consists of 19 questions about DSM-5 symptoms of PMDD and their impact on various domains of functioning, responding on a four-point Likert-type scale ranging from “not at all” to “severe”. The scale enables diagnosis of PMDD (at least one “severe” core emotional symptom, at least five symptoms “moderate to severe”, at least one domain of functioning is “severely” impacted) and moderate to severe PMS (the same criteria but with “moderate to severe” intensity). For this study additional distinction between severe (7 or more moderate to severe symptoms) and moderate PMS was done. It is, however, important to note that PSST is a retrospective measure, thus the diagnosis of any premenstrual disorder is not definite, but rather informs about the number and intensity of the premenstrual symptoms. The Cronbach’s alfa for the PSST in the sample was 0.93.

Depressive symptoms were measured with the Polish version of Beck Depression Inventory Second Edition (BDI-II)^36,37^. The BDI-II consists of 21 items to which participants answer using a four-point scale. The Cronbach’s alfa in the sample was 0.91.

The level of anxiety was assessed with the Polish version of State-Trait Anxiety Inventory (STAI)^38^. The STAI consist of 40 items and two subscales: state anxiety and trait anxiety. Participants answer each question using a four-point Likert-like scale. The state subscale was not included in analyses for two reasons: first, the hypotheses concerned the trait anxiety as a more stable tendency to worry and feel anxious, second, the study was conducted not long after the Russian invasion on Ukraine, which is a neighbor of Poland, therefore some participants may have felt more anxious during this time. This applies especially to people examined in the earlier stage of the study. The Cronbach’s alfa for the trait scale was 0.93.

Psychotic-like experiences were measured with the Polish version of the Community Assessment of Psychic Experiences (CAPE)^28,39^. The CAPE consists of 42 questions answered with a four-point Likert-like scale marking the frequency of psychotic-like experiences. The CAPE is divided into three subscales measuring negative (14 items) and positive (20 items) PLEs, and depressive symptoms (8 items). Additionally, participants mark distress related to every symptom they experience. However in the study, the distress scale was not used, since the hypotheses considered only the frequency of PLEs. Depressive symptoms scale was also not taken into account, as it would overlap with BDI-II scores. The Cronbach’s alphas in the study sample were: for the positive subscale *α* = 0.85 and for the negative subscale *α* = 0.86.

All copyrighted instruments were used with the permission of the copyright holders.

Participants were recruited during their university classes. They were informed about the aim of the study, time needed to complete it, and their rights; all their questions were addressed. All participants were volunteers and did not receive compensation. The study was conducted in accordance with the Declaration of Helsinki. Informed, written consent was obtained from all participants. The study was approved by the Jagiellonian University Institute of Psychology Research Ethics Committee (KE/9_2022).

### Analysis plan

To test the study hypotheses, the correlation, regression, and mediation analyses were run. For mediation analysis the following conditions were used as suggested by MacKinnon^40^: (1) significant association between premenstrual symptoms and PLEs; (2) significant association between mediators (depressive symptoms and anxiety) and PLEs while premenstrual symptoms were controlled; (3) a significant coefficient for the indirect path between premenstrual symptoms and PLEs through mediators. The differences between groups (‘no diagnosis’, ‘moderate PMS’, ‘severe PMS’, and ‘PMDD’) were also tested. The IBM SPSS Statistics for Windows version 28.0 and PROCESS package were used.

## Results

The statistics for study variables are presented in Table 1. Four groups were distinguished regarding PSST scores: a ‘no diagnosis’ group (*n* = 44; 40.7%), a ‘moderate PMS’ group (*n* = 14; 13%), a ‘severe PMS’ group (*n* = 21; 19.4%), and a ‘PMDD’ group (*n* = 29; 26.9%). Statistics for other study variables in premenstrual symptoms groups are presented in Table 2. According to Shapiro–Wilk normality test, most variables did not have normal distribution, trait anxiety being the only exception (*p* = 0.146). Thus, non-parametric test were performed.

**Table 1:** Statistics of the study variables.

	Mean	SD	Median	Min	Max
PSST total	42.53	12.88	45.00	19	71
BDI II	13.57	9.58	10.00	0	44
STAI trait	45.97	11.98	47.00	20	72
CAPE positive	34.60	7.74	34.00	23	63
CAPE negative	28.09	8.35	28.00	14	55

Statistics of study variables, N = 108.

*PSST* Premenstrual Symptoms Screening Tool, *BDI II* Beck Depression Inventory 2nd Edition, *STAI* State-Trait Anxiety Inventory, *CAPE* Community Assessment of Psychic Experiences.

**Table 2:** Statistics of the study variables in premenstrual diagnosis groups.

	No diagnosis M (*SD*)	Moderate PMS M (*SD*)	Severe PMS M (*SD*)	PMDD M (*SD*)
BDI II	7.55 (4.71) *Mdn* = 7.00	13.86 (8.25) *Mdn* = 12.50	17.00 (8.99) *Mdn* = 14.00	20.10 (10.83) *Mdn* = 19.00
STAI trait	38.34 (9.82) *Mdn* = 38.50	47.57 (7.63) *Mdn* = 48.00	50.71 (11.15) *Mdn* = 51.00	53.35 (10.92) *Mdn* = 55.00
CAPE positive	29.43 (4.13) *Mdn* = 28.00	33.79 (5.18) *Mdn* = 34.50	38.00 (*SD* = 6.95) *Mdn* = 40.00	40.38 (8.38) *Mdn* = 39.00
CAPE negative	22.66 (6.47) *Mdn* = 20.00	28.93 (4.32) *Mdn* = 28.50	31.38 (*SD* = 7.90) *Mdn* = 31.00	33.55 (7.96) *Mdn* = 33.00

Overall N = 108, N_no diagnosis_ = 44 (40.7%); N_moderate PMS_ = 14 (13%); N_severe PMS_ = 21 (19.4%); N_PMDD_ = 29 (26.9%).

*M* mean, *Mdn* median, *BDI II* Beck Depression Inventory 2nd Edition, *STAI* State-Trait Anxiety Inventory, *CAPE* Community Assessment of Psychic Experiences.

### The relationship between premenstrual symptoms, psychotic-like experiences, depression, and anxiety

Correlation analysis were done with Spearman’s *rho* test. All correlations between study variables were significant (see Table 3).

**Table 3:** Correlations between study variables (Spearman’s *rho*).

	PSST	BDI II	STAI trait	CAPE positive	CAPE negative
PSST	–				
BDI II	0.62***	–			
STAI trait	0.59***	0.82***	–		
CAPE positive	0.65***	0.54***	0.54***	–	
CAPE negative	0.64***	0.77***	0.83***	0.64***	–

Correlation was calculated with Spearman’s *rho*; the assumed level of statistical significance was *α* ≤ 0.05.

*PSST* Premenstrual Symptoms Screening Tool, *BDI II* Beck Depression Inventory 2nd Edition, *STAI* State-Trait Anxiety Inventory, *CAPE* Community Assessment of Psychic Experiences.

***Statistically significant, *p* < 0.001.

### Between groups differences in depression, anxiety, and PLEs frequency

Levels of depressive symptoms, trait anxiety, and PLEs frequency were compared between the ‘no diagnosis’, ‘moderate PMS’, ‘severe PMS’, and ‘PMDD’ groups. The Kruskal–Wallis test and pairwise comparisons with Bonferroni-adjustment were performed.

The level of depressive symptoms differed significantly between the groups (*H*(3) = 33.87, *p* < 0.001): BDI-II scores median in the ‘no diagnosis group’ were significantly lower than in the ‘moderate PMS’ group (*p* = 0.013), and other groups (*p* < 0.001).

Trait anxiety level also differed significantly between groups (*H*(3) = 22.40, *p* < 0.001): the ‘no diagnosis’ group scored lower than the ‘severe PMS’ (*p* = 0.009) and the ‘PMDD’ (*p* < 0.001) groups.

Positive PLEs frequency differed significantly between groups (*H*(3) = 41.26, *p* < 0.001): the ‘no diagnosis group’ scored significantly lower than the ‘moderate PMS’ (*p* < 0.035) and other groups (*p* < 0.001), the ‘moderate PMS’ group experienced less positive PLEs than the ‘PMDD group’ (*p* = 0.019). Negative PLEs also differed significantly between groups (*H*(3) = 26.32, *p* < 0.001): the ‘no diagnosis’ group scored lower than the ‘moderate PMS’ (*p* = 0.004), ‘severe PMS’ (*p* = 0.002), and ‘PMDD’ groups (*p* < 0.001). None of the other comparisons were significant.

### Premenstrual symptoms, depression, and anxiety as predictors of positive and negative PLEs frequency

Regression analysis was performed separately for negative and positive PLEs. The hierarchical model was used with premenstrual symptoms severity as a predictor in the first step, depressive symptoms and trait anxiety were added as control variables in the second step. In all cases, the assumptions of normality and homoscedasticity of residuals were met. The tolerance and VIF coefficients suggested some collinearity between depressive symptoms and trait anxiety (but not premenstrual symptoms). All predictors were added manually to the model, with the authors determining the order in which they were entered.

#### Model 1

First, a model for positive PLEs frequency was tested. It was significant (*F*(1,106) = 77.09; *p* < 0.001) and explained about 42% in the dependent variable variance (adjusted determinant coefficient). The Durbin–Watson statistic was good (1.85). Premenstrual symptoms were a significant predictor of positive PLEs frequency (*p* < 0.001; 42% of variance explained). When depressive symptoms and trait anxiety were added in the second step, the model remained significant (*F*(3,104) = 28.50; *p* < 0.001), explaining about 44% in the variance of positive psychotic-like experiences. Premenstrual symptoms were the only non-redundant predictor of the dependent variable (*p* < 0.001), explaining about 17% of its variance. The detailed results can be seen in Table 4.

**Table 4:** Multiple regression analysis with premenstrual symptoms, depressive symptoms and trait anxiety on positive PLEs frequency.

Predictors	*B*	*Beta*	*r*_*semi*_	% of variance	*t*	*p*
Step 1						
(Constant)	18.02 (95% CI 14.11 to 21.93)				9.14	< 0.001
Premestrual symptoms	0.39 (95% CI 0.30 to 0.48)	0.65	0.65	42	8.78	** < 0.001**
Step 2						
(Constant)	15.85 (95% CI 10.12 to 21.59)				5.48	< 0.001
Premenstrual symptoms	0.31 (95% CI 0.20 to 0.42)	0.51	0.41	17	5.57	** < 0.001**
Depressive symptoms	0.05 (95% CI − 0.15 to 0.25)	0.06	0.04	< 1	0.52	0.602
Trait anxiety	0.11 (95% CI − 0.05 to 0.27)	0.17	0.10	1	1.33	0.188

The hierarchical model with premenstrual symptoms severity as a predictor in the first step, depressive symptoms and trait anxiety were added in the second step. 95% confidence intervals are showed in brackets.

*r*_*semi*_ semi-partial correlation coefficient, *% of variance* the percent of the variance of dependent variable explained by the predictor.

Significant predictors are in bold.

#### Model 2

The next model tested predictors of negative PLEs frequency. In the first step, the model was significant (*F*(1,106) = 71.45; *p* < 0.001) and explained about 40% of the variance of dependent variable. Durbin-Watson statistic was acceptable (1.82). Premenstrual symptoms were a significant predictor of negative PLEs frequency (*p* < 0.001; almost 41% of variance explained). In the second step the model remained significant (*F*(3,104) = 82.14; *p* < 0.001) and explained 70% of the dependent variable variance. Premenstrual symptoms (3% of variance explained; *p* = 0.003), depressive symptoms (2% of variance explained; *p* = 0.016) and trait anxiety (8% of variance explained; *p* < 0.001) were all non-redundant predictors of negative PLEs frequency. The detailed results are presented in Table 5.

**Table 5:** Multiple regression analysis with premenstrual symptoms, depressive symptoms and trait anxiety on negative PLEs frequency.

Predictors	*B*	*Beta*	*r*_*semi*_	% of variance	*t*	*p*
Step 1						
(Constant)	10.60 (95% CI 6.31 to 14.88)				4.90	< 0.001
Premenstrual symptoms	0.41 (95% CI 0.32 to 0.51)	0.64	0.64	41	8.45	** < 0.001**
Step 2						
(Constant)	3.54 (95% CI − 1.01 to 8.09)				1.54	0.126
Premenstrual symptoms	0.14 (95% CI 0.05 to 0.22)	0.21	0.16	3	3.06	**0.003**
Depressive symptoms	0.19 (95% CI 0.04 to 0.35)	0.22	0.13	2	2.45	**0.016**
Trait anxiety	0.35 (95% CI 0.23 to 0.48)	0.51	0.29	8	5.51	** < 0.001**

The hierarchical model with premenstrual symptoms severity as a predictor in the first step, depressive symptoms and trait anxiety were added in the second step. 95% confidence intervals are showed in brackets.

*r*_*semi*_ semi-partial correlation coefficient, *% of variance* the percent of the variance of dependent variable explained by the predictor.

Significant predictors are in bold.

### Mediation of the relationship between premenstrual symptoms and PLEs through depression and anxiety

Next, mediation analysis was performed, separately with depressive symptoms and trait anxiety as mediators of the relationship between the symptoms of premenstrual disorders and PLEs. The indirect effects significance was calculated with bootstrap method (5000 samples) for 95% confidence intervals.

#### Depressive symptoms as mediators

**Figure 1:**
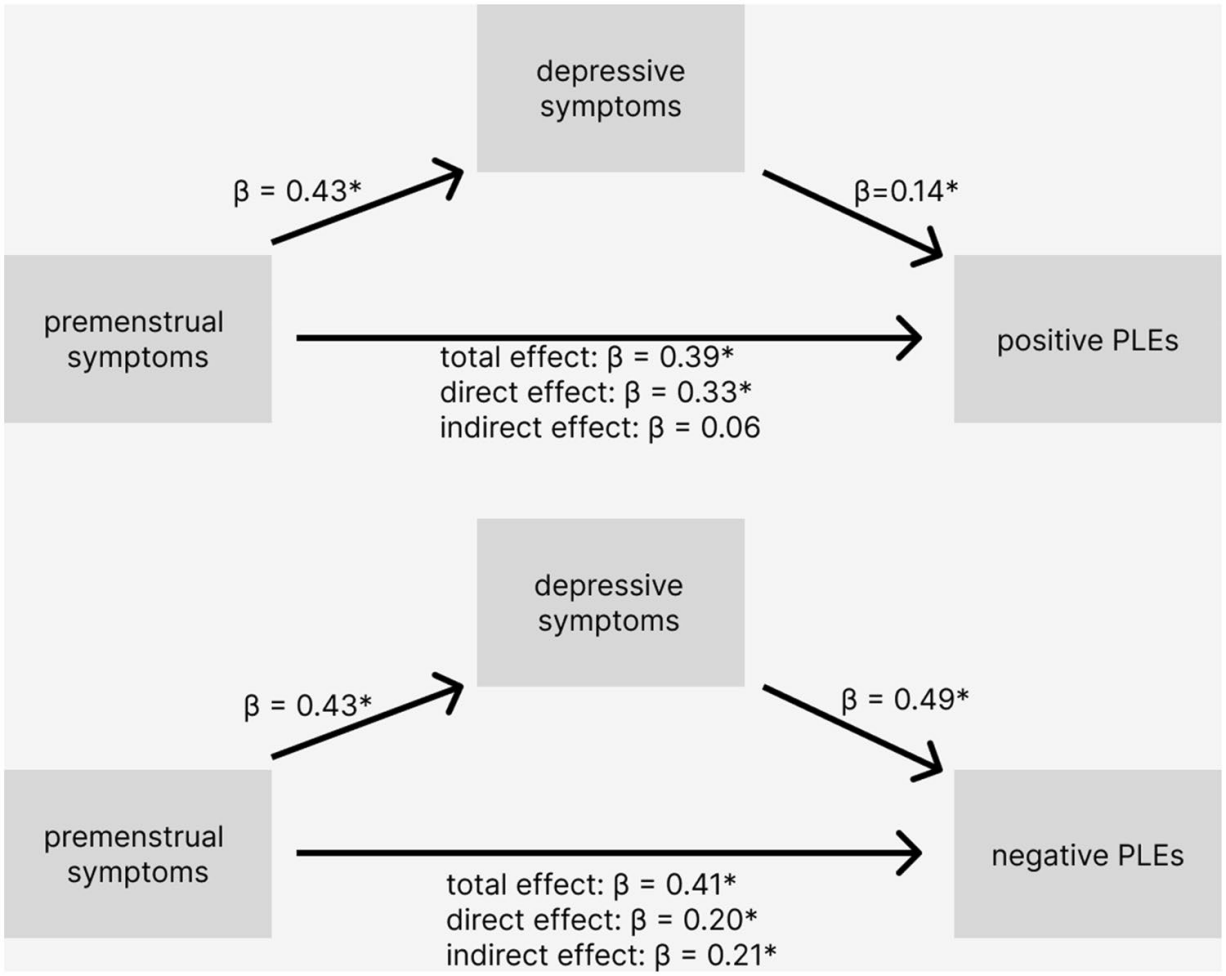
Depressive symptoms as a mediator of the relationship between premenstrual symptoms and psychotic-like experiences. Diagram shows the mediation of the relationship between the retrospectively measured premenstrual symptoms and the frequency of positive (upper) and negative (bottom) psychotic-like experiences through depressive symptoms. The beta values are not standardized. Predictions significant at α ≤ 0.05 are marked with asterisks.

Mediation through the depressive symptoms is visualised in Fig. 1. Premenstrual symptoms predicted depressive symptoms (*β* = 0.43, 95% CI 0.31–0.54; *p* < 0.001) and depressive symptoms significantly predicted positive (*β* = 0.14; *p* = 0.050) and negative PLEs frequency (*β* = 0.49, 95% CI 0.36–0.62; *p* < 0.001). Total effects of premenstrual symptoms on positive (*β* = 0.39, 95% CI 0.30–0.48; *p* < 0.001) and negative PLEs (*β* = 0.41, 95% CI 0.32–0.51; *p* < 0.001) were significant. Direct effects were also significant but in case of positive PLEs, very similar to the total effect (*β* = 0.33; 95% CI 0.22–0.44; *p* < 0.001); for negative PLEs the direct effect was weaker than the total effect (*β* = 0.20, 95% CI 0.11–0.30; *p* < 0.001). The indirect effect of premenstrual symptoms on positive PLEs frequency through depressive symptoms was not significant (*β* = 0.06, 95% CI − 0.01 to 0.14). The indirect effect for negative PLEs (*β* = 0.21, 95% CI 0.13–0.32) was significant which suggests partial mediation in their case.

#### Trait anxiety as mediator

**Figure 2:**
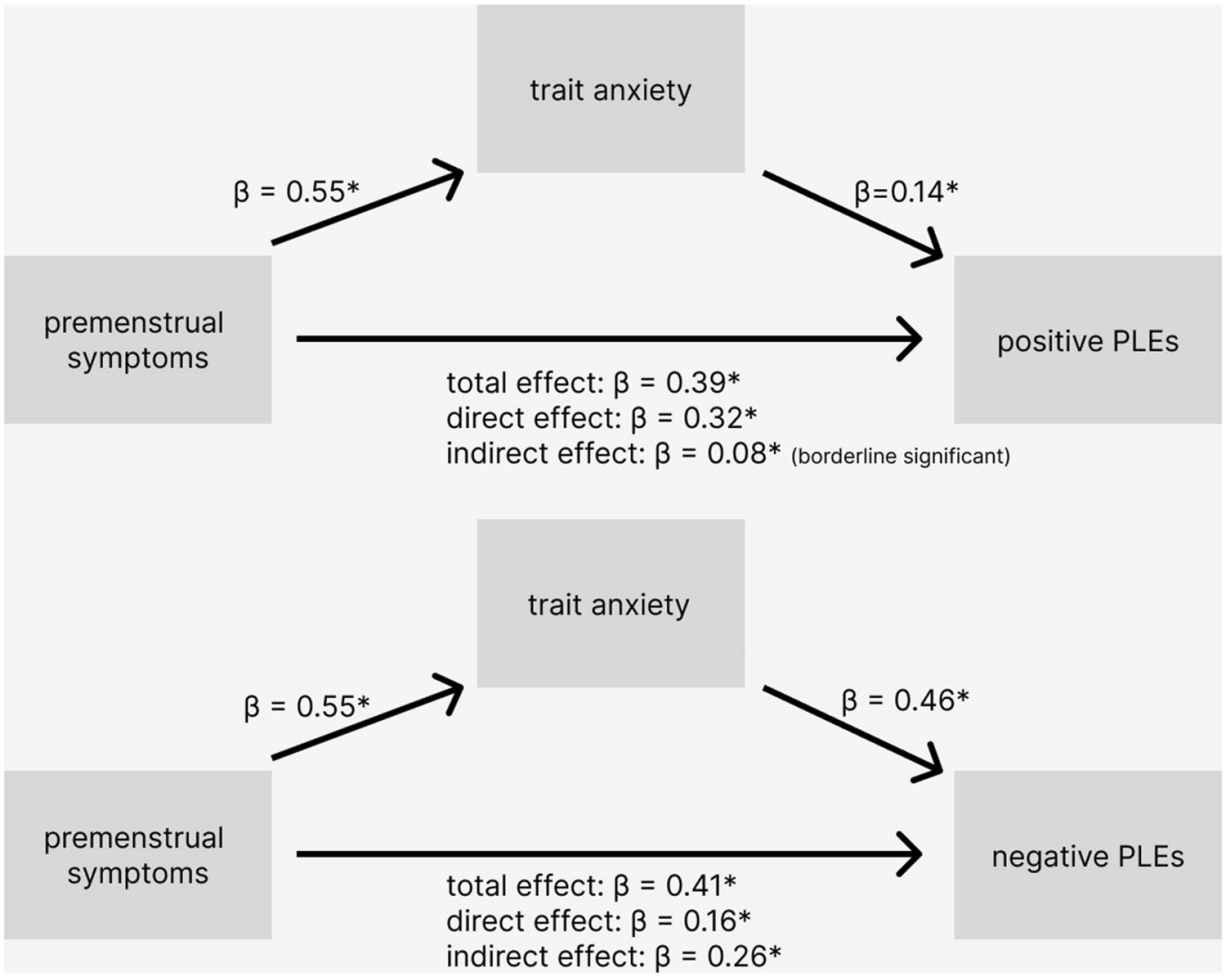
Trait anxiety as a mediator of the relationship between premenstrual symptoms and psychotic-like experiences. Diagram shows the mediation of the relationship between the retrospectively measured premenstrual symptoms and the frequency of positive (upper) and negative (bottom) psychotic-like experiences through trait anxiety. The beta values are not standardized. Predictions significant at α ≤ 0.05 are marked with asterisks.

Mediation through trait anxiety is visualised in Fig. 2. Premenstrual symptoms successfully predicted trait anxiety level (*β* = 0.55, 95% CI 0.49–0.70 *p* < 0.001) and trait anxiety successfully predicted positive (*β* = 0.14; 95% CI 0.02–025; *p* < 0.001) and negative PLEs frequency (*β* = 0.46, 95% CI 0.38–0.66; *p* < 0.001). Total effects of premenstrual symptoms on positive PLEs (*β* = 0.39, 95% CI 0.30–0.48; *p* < 0.001) and negative PLEs (*β* = 0.41, 95% CI 0.32–0.51; *p* < 0.001) were significant. Direct effects were also significant but in case of positive PLEs, similar to the total effect (*β* = 0.32; 95% CI 0.21–0.42; *p* < 0.001); for negative PLEs the direct effect was weaker than the total effect (*β* = 0.16, 95% CI 0.07–0.24; *p* < 0.001). The indirect effect of premenstrual symptoms on positive PLEs frequency through trait anxiety was on the verge of significance (*β* = 0.08, 95% CI 0.01–0.14). Indirect effect for negative PLEs (*β* = 0.26, 95% CI 0.18–0.34) was also significant, which suggests partial mediation.

## Discussion

In the presented study we examined the relationship between the subjective, retrospectively measured intensity of the symptoms of premenstrual disorders and psychotic-like experiences. We also tested if depressive symptoms and trait anxiety mediate said relationship. Premenstrual symptoms indeed predicted psychotic-like experiences frequency in the regression analysis. Depression and anxiety partially mediated this relationship for negative but not positive PLEs.

The prevalence of PMS (32.4%) in the study sample was similar to the estimated population prevalence (20–32%)^5^ while PMDD was much more frequent than in the general population (26.9% vs 3–8%). While over-detection of premenstrual disorders may be one of the weaknesses of retrospective measures, studies suggest that PSST allows to identify people with premenstrual disorders but fails to accurately differentiate between PMS and PMDD^41,42^. Some studies also suggest that premenstrual disorders may be more common in university students population (e.g. 7.7% prevalence of PMDD and 92.3% prevalence of PMS in the study of Hussein-Shehadeh and Hamdan-Mansour^43^ or 10.2% prevalence of PMDD in study of Roomaney and Lourens^44^—it is important to note however, that the latter also used retrospective measure). Additionally, the study was performed during the COVID-19 pandemic. Some research indicate that premenstrual disorders prevalence and severity increased during the pandemic^45^. Lastly, the study was performed not long after the Russian aggression on Ukraine began. As Ukraine is a neighbouring country of Poland, this could have been a severe stressor for some participants, which thus influenced the severity of their premenstrual symptoms. Indeed, increased stress have been shown to be a risk factor for PMS and PMDD development^46^. All these factors could have contributed to higher than estimated population prevalence of premenstrual disorders in the sample—but of course do not exclude the probability of the over-detection of PMS and PMDD by PSST.

Between-group comparisons showed that people reporting to have very mild premenstrual symptoms significantly differed in the levels of depression, anxiety, positive and negative PLEs from those who declared to experience more severe premenstrual symptoms. This finding supports the hypothesis that subjective intensity of premenstrual symptoms may be an important factor related to psychotic symptomatology. Lack of differences between the three premenstrual disorders groups is probably related to low specificity and discrimination abilities of a retrospective measure. The other possible explanation is that premenstrual symptomatology takes a form of a continuum rather than of separate diagnostic categories.

As expected, the retrospectively measured symptoms of premenstrual disorders were significantly, positively correlated with PLEs. Moreover, premenstrual symptoms were significant predictors of PLEs, both positive and negative subtypes, also while depressive symptoms and trait anxiety were controlled. That relationship was especially visible for positive PLEs, for which premenstrual symptoms were the strongest correlate and the best (and only non-redundant) predictor. This strong relation probably mirrors the gender differences in PLEs frequency: more positive PLEs in women than in men^12^, and is consistent with the results of the Hsiao and Liu study^24^ showing that positive psychotic symptoms can be unusual manifestations of premenstrual disorders.

The mediation analyses revealed that depression and anxiety partially mediate the relationship between the retrospectively measured premenstrual symptoms and negative PLEs. This is not surprising as depression and anxiety were shown to be related to premenstrual disorders, psychosis, and psychotic-like experiences^16,47^. Negative PLEs consist of such symptoms as apathy, social withdrawal, limitation of activity. Increased depression and anxiety may reduce the motivation for engaging in social life and usual activities, especially when the anxiety has social component as in case of socially stigmatized disorder such as premenstrual disorders^48^. This result thus suggests that the relationship between premenstrual symptoms and negative PLEs is—at least partially—based on a shared, general psychopathological factor. The mediation was however only partial, which shows that premenstrual disturbances are indeed an important factor related to PLEs which cannot be reduced only to general proneness to mental disorders.

Depressive symptoms did not mediate the relationship between retrospectively measured premenstrual symptoms and positive PLEs (delusions-like and hallucinations-like experiences). For the same relationship, anxiety was a very weak and borderline significant mediator. In the Hsiao and Liu^24^ study delusions and hallucinations were observed in women suffering from PMS. In the current study premenstrual symptoms were the strongest correlate and best predictor of positive PLEs. Hence, the relationship between premenstrual symptoms and positive PLEs may be considered as a direct one, not mediated through other symptoms.

There can of course be other mediators of the relationship between premenstrual symptoms and delusion-like and hallucinations-like experiences, for example psychotic cognitive biases. There is some evidence that women with premenstrual disorders have tendency for cognitive biases typical of depression^49^. However, the prevalence of psychosis-related biases such as attention to threat or jumping to conclusions in PMS and PMDD were not examined.

It is important to note that the current study was based on retrospective data. As premenstrual disorders require prospective diagnosis^3^, using PSST do not provide a definite diagnosis of either PMS or PMDD. PSST scores rather inform about the presence of some symptoms or can be used as a measure of a person’s own evaluation of these symptoms. Thus the results of this study can also indicate that psychotic-like experiences are related rather to the person’s beliefs about own menstruation and premenstrual symptoms. In that light, the stronger relationship between PSST scores and positive PLEs might be also based on some cognitive distortions. Positive PLEs include delusion-like beliefs and retrospective measures of premenstrual symptoms are influenced by the beliefs about menstruation^50^ or even internalized misogyny^51^. This is yet another argument on cognitive biases being possible mediators of the relationship between premenstrual symptoms and positive PLEs.

### Limitations

This study was not free of limitations. The sample was relatively small and limited to university students. While young adults were shown to present higher levels of both PLEs^13,19^ and premenstrual disorders^43,44^, it may not be representative for the general population. Secondly, the time the study was performed might have influenced the results. Finally, only retrospective measurements were implemented and the study was cross-sectional, thus it is impossible to infer causality. This also resulted in a probable over-detection of premenstrual disorders prevalence in the sample. Although the retrospective measurements are widely used in premenstrual disorders studies (e.g.^44,52^), they are not considered a fully reliable measure for their diagnosis^53^. It is more appropriate to interpret the scores of PSST questionnaire as a person’s beliefs about own symptoms rather than the definite diagnosis of premenstrual disorders.

Moreover, the exclusion criteria were evaluated by self-reports; there is possibility that some participants did not admit to taking psychoactive substances or were not diagnosed yet (but had some mental or neurological disorders). They could have also failed to differentiate their depressive symptoms from both PMS/PMDD symptoms and negative PLEs (some participants might, for instance, have mistaken anhedonia resulting from their undiagnosed affective disorder as emotional blunting, which is an example of negative psychotic symptom). Some of the participants might have also counted their “usual” psychopathological symptoms (e.g., anxiety, depressive, or externalising symptoms) as premenstrual (premenstrual tension, sadness or irritability) and thus admit to have higher scores on PSST. Generally speaking, the usage of retrospective measures is unfortunately susceptible to bias. Finally, some collinearity between depressive symptoms and trait anxiety as predictors in the regression analysis inclines to caution in its interpretation.

## Conclusions

To our knowledge, this is the first study to examine the relationship between premenstrual symptoms and subclinical psychotic symptoms. Despite limitations, this study demonstrates strong, positive relationship between retrospectively measured symptoms of premenstrual disorders and psychotic-like experiences. It is important to note that even though the study was based on retrospective measure, which does not allow definite diagnosis, Wittchen et al.^47^ demonstrated that approximately 19% of naturally-cycling individuals may be affected by subclinical PMDD in which not all diagnostic criteria are met, but a significant functional impairment is present. In other words, testing the whole continuum of premenstrual symptomatology, including subclinical states or people’s beliefs about own symptoms, may be valuable for better understanding menstruating people’s cyclical psychological changes.

However, more research is needed on the topic—preferably with prospective measure of premenstrual symptomatology and on other populations. Additionally, possible moderators of the relationship between premenstrual symptoms and PLEs, and protective factors, preventing from developing PLEs in menstruating women should be sought in future studies.

Nevertheless, the results suggest that subclinical psychotic symptoms may indeed be a manifestation of premenstrual symptomatology—which is consistent with results of Hsiao and Liu’s^24^ case study—or that premenstrual disturbances increase risk of developing psychotic symptoms (or at least their subclinical forms). Moreover the relationship between premenstrual symptoms and positive psychotic-like experiences, was not explained by depressive symptoms or anxiety. This suggests that premenstrual symptomatology is somehow especially related to proneness to positive psychotic symptoms—which are also the symptoms more common among women^9,12,13^. One possible explanation of that relationship are fluctuations in the reproductive hormones levels and increased sensitivity to these fluctuations, which is considered one of the most probable etiological factors of PMDD^54^.

On the other hand, the results may also suggest that higher levels of PLEs may also constitute a risk factor of developing premenstrual disorders. Psychotic-like experiences were indeed shown to increase the risk of developing many mental disorders^19^ and premenstrual disorders might be among them.

## Data Availability

All data associated with this research may be assessed via e-mail to corresponding author. Data is not stored in open-access database to protect participants’ from accidental violation of confidentiality.
